# Crystal structure of oxam­yl

**DOI:** 10.1107/S2056989016018168

**Published:** 2016-11-18

**Authors:** Eunjin Kwon, Ki-Min Park, Hyunjin Park, Tae Ho Kim

**Affiliations:** a Department of Chemistry (BK21 plus) and Research Institute of Natural Sciences, Gyeongsang National University, Jinju 52828, Republic of Korea

**Keywords:** crystal structure, acaricide, insecticide, nematicide, oxam­yl

## Abstract

The title compound, C_7_H_13_N_3_O_3_S [systematic name: (*Z*)-methyl 2-di­methyl­amino-*N*-(methyl­carbamo­yloxy)-2-oxoethanimido­thio­ate], is an oxime carbamate acaride, insecticide and nematicide. The asymmetric unit comprises two independent mol­ecules, *A* and *B*. The dihedral angles between the mean planes [r.m.s. deviations = 0.0017 (*A*) and 0.0016 Å (*B*)] of the acetamide and oxyimino groups are 88.80 (8)° for *A* and 87.05 (8)° for *B*. In the crystal, N/C—H⋯O hydrogen bonds link adjacent mol­ecules, forming chains along the *a* axis. The chains are further linked by C—H⋯O hydrogen bonds, resulting in a three-dimensional network with alternating rows of *A* and *B* mol­ecules in the *bc* plane stacked along the *a*-axis direction. The structure was refined as an inversion twin with a final BASF parameter of 0.16 (9).

## Abstract 2

The title compound, which is used as an acaricide, insecticide and mematicide, crystallizes with two independent mol­ecules in the asymmetric unit. In the crystal, hydrogen bonds link adjacent mol­ecules, forming a three-dimensional network of mol­ecules stacked along the *a*-axis direction.

## Chemical context   

Oxamyl [(*N*,*N*-dimethyl-2-methyl­carbamoyloximino-2-(di­methyl­sulfan­yl)acetamide] is a carbamate compound used in a wide range of agricultural situations. It is systemic and active as an insecticide or a nematicide. It is used for the control of nematodes in vegetables, bananas, pineapple, peanuts, cotton, soya beans, tobacco, potatoes, sugar beet, and other crops. It is also used in India for controlling the growth of nematodes on vegetable crops (Mohammad *et al.*, 2015 ▸; Agarwal *et al.*, 2016 ▸). In addition, oxamyl was classified by the World Health Organization (WHO) as highly haza­rdous (class IB) (Al-Dabbas *et al.*, 2014 ▸). Oxamyl can be integrated with horse manure, sesame-oil-cake, or *Bacillus thuringiensis* to improve eggplant growth response and reduce development of the nematode *Meloidogyne incognita* (Osman *et al.*, 2009 ▸). Also, oxamyl has a very high water solubility (280 g/L at 298 K) and low sorption solubility affinity to soils. As a result of these properties, oxamyl easily migrates into the water compartment (Mazellier *et al.*, 2010 ▸). Herein, we report the mol­ecular and crystal structure of oxamyl.
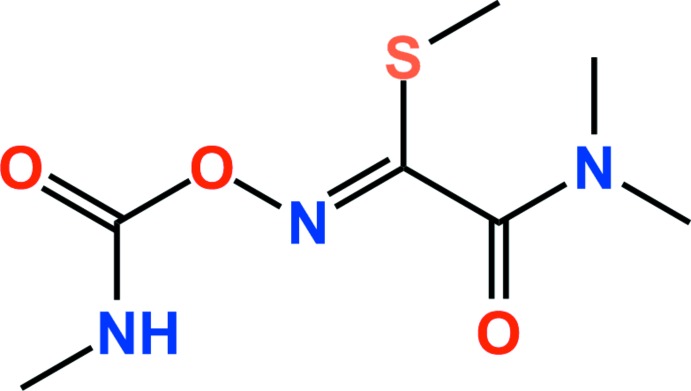


## Structural commentary   

**Figure 1:**
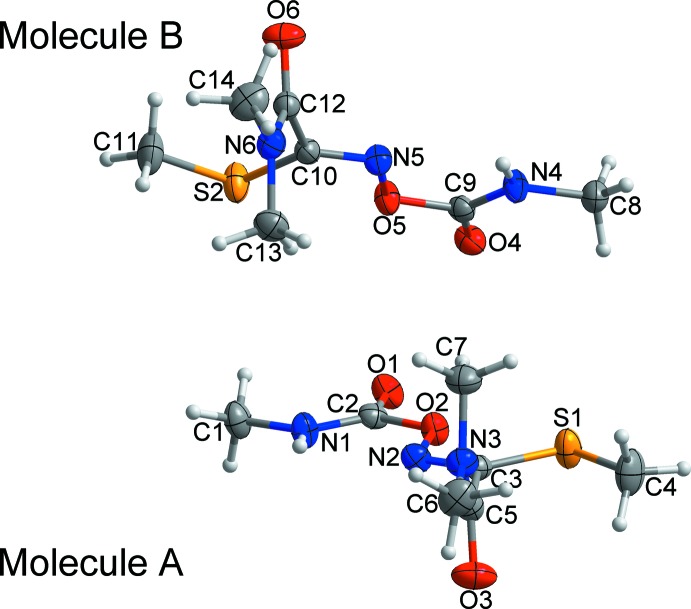
The asymmetric unit of the title compound, showing the atom-numbering scheme. Displacement ellipsoids are drawn at the 50% probability level. H atoms are shown as small spheres of arbitrary radius.

The asymmetric unit of oxamyl comprises two independent mol­ecules, *A* and *B* (Fig. 1 ▸). The compound consists of carbamate, acetamide, methyl­thio and oxyimino functional groups. The dihedral angles between the mean planes [r.m.s. deviations = 0.0017 (*A*) and 0.0016 Å (*B*)] of the acetamide and oxyimino groups are 88.80 (8) for *A* and 87.05 (8)° for *B*. All bond lengths and bond angles are normal and comparable to those observed in methomyl [systematic name: (*E*)-methyl *N*-(methyl­carbamo­yl)oxyethanimido­thio­ate] which adopts similar crystal structure (Takusagawa & Jacobson, 1977 ▸).

## Supra­molecular features   

**Figure 2:**
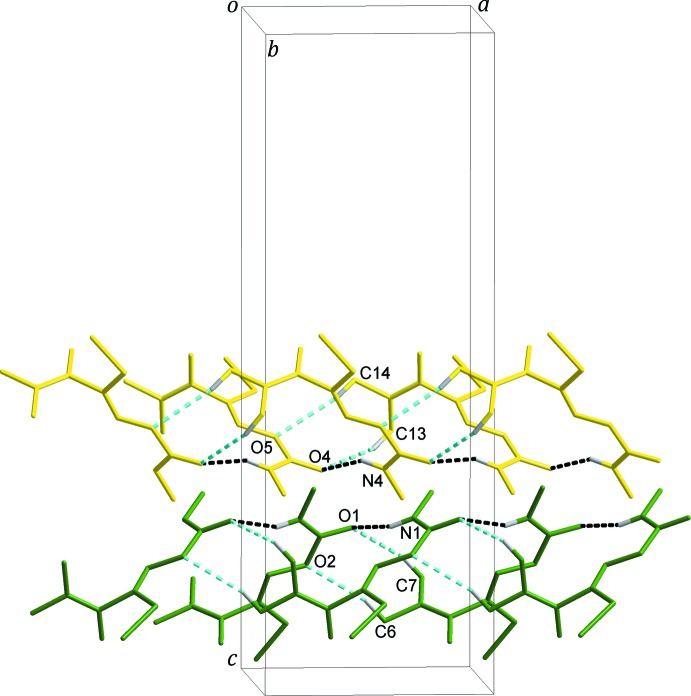
The double chains formed through inter­molecular N—H⋯O (black dashed lines) and C—H⋯O (sky-blue dashed lines) hydrogen bonds. The *A* and *B* mol­ecules are shown in green and yellow, respectively. H atoms not involved in inter­molecular inter­actions have been omitted for clarity.

**Figure 3:**
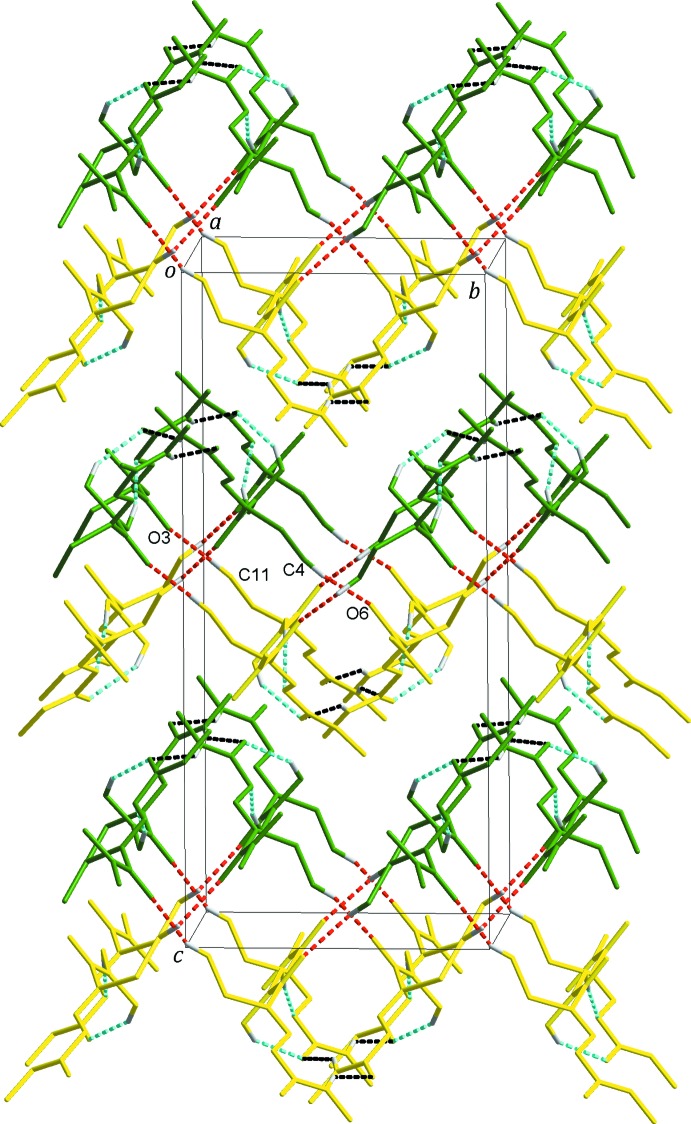
The three-dimensional network made up of mol­ecules *A* (green) and *B* (yellow). Black dashed lines represent inter­molecular N—H⋯O hydrogen bonds. The C—H⋯O hydrogen bonds are shown as sky-blue (between each mol­ecule *A* or *B*) and red (between mol­ecules *A* and *B*) dashed lines, respectively. H atoms not involved in inter­molecular inter­actions have been omitted for clarity.

The crystal structure is stabilized by several N—H⋯O and C—H⋯O hydrogen bonds (Table 1 ▸). Adjacent *A* mol­ecules form inter­molecular N1—H1*N*⋯O1 hydrogen bonds. In addition, C6—H6*B*⋯O2 and C7—H7*B*⋯O1 hydrogen bonds between the carbamate and di­methyl­amine groups generate 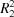
(8) inversion dimers. These contacts link the *A* mol­ecules into double chains along the *a* axis. A closely similar situation obtains for the *B* mol­ecules, with inter­molecular N4—H4*N*⋯O4 hydrogen bonds together with C13—H13*B*⋯O4 and C14—H14*B*⋯O5 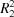
(8) inversion dimers also forming a double chain, this time solely of *B* mol­ecules, parallel to the one described previously, again along the *a* axis, Fig. 2 ▸. The *A* and *B* double chains are further linked by C4—H4*B*⋯O6 and C11—H11*B*⋯O3 contacts, Table 1 ▸, to give a three-dimensional network with alternating rows of *A* and *B* mol­ecules in the *bc* plane stacked along the *a*-axis direction, Fig. 3 ▸.

## Synthesis and crystallization   

The title compound was purchased from Dr Ehrenstorfer GmbH. Slow evaporation of its solution in CH_3_OH gave single crystals suitable for X-ray analysis.

## Refinement   

Crystal data, data collection and structure refinement details are summarized in Table 2 ▸. All C-bound H atoms were positioned geometrically [with *d*(N—H) = 0.88 Å, *U*_iso_ = 1.2*U*_eq_(C) for N—H group, *U*_iso_ = 1.5*U*_eq_(C) for methyl group, *d*(C—H) = 0.98 Å]. The crystal studied was an inversion twin with a 0.84 (9):0.16 (9) domain ratio.

## Supplementary Material

Crystal structure: contains datablock(s) I, New_Global_Publ_Block. DOI: 10.1107/S2056989016018168/sj5514sup1.cif

Structure factors: contains datablock(s) I. DOI: 10.1107/S2056989016018168/sj5514Isup2.hkl

Supporting information file. DOI: 10.1107/S2056989016018168/sj5514Isup3.cml

CCDC reference: 1516996

Additional supporting information: crystallographic information; 3D view; checkCIF report

## Figures and Tables

**Table 1:** Hydrogen-bond geometry (Å, °)

*D*—H⋯*A*	*D*—H	H⋯*A*	*D*⋯*A*	*D*—H⋯*A*
N1—H1*N*⋯O1^i^	0.88	2.13	2.871 (3)	142
N4—H4*N*⋯O4^ii^	0.88	2.04	2.794 (3)	142
C4—H4*B*⋯O6^iii^	0.98	2.54	3.075 (4)	114
C6—H6*B*⋯O2^iv^	0.98	2.60	3.518 (4)	156
C7—H7*B*⋯O1^iv^	0.98	2.52	3.431 (4)	155
C11—H11*B*⋯O3^v^	0.98	2.53	3.042 (4)	113
C13—H13*B*⋯O4^iv^	0.98	2.53	3.440 (4)	155
C14—H14*B*⋯O5^iv^	0.98	2.59	3.554 (4)	168

Symmetry codes: (i) 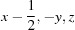; (ii) 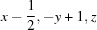; (iii) 
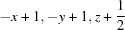
; (iv) 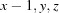; (v) 
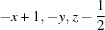
.

**Table 2:** Experimental details

Crystal data	
Chemical formula	C_7_H_13_N_3_O_3_S
*M*_r_	219.26
Crystal system, space group	Orthorhombic, *P* *c* *a*2_1_
Temperature (K)	173
*a*, *b*, *c* (Å)	8.3367 (4), 10.7752 (5), 24.1016 (12)
*V* (Å^3^)	2165.04 (18)
*Z*	8
Radiation type	Mo *K*α
μ (mm^−1^)	0.29
Crystal size (mm)	0.50 × 0.14 × 0.11

Data collection	
Diffractometer	Bruker APEXII CCD
Absorption correction	Multi-scan (*SADABS*; Bruker, 2014 ▸)
*T*_min_, *T*_max_	0.665, 0.746
No. of measured, independent and observed [*I* > 2σ(*I*)] reflections	19300, 5238, 4655
*R*_int_	0.035
(sin θ/λ)_max_ (Å^−1^)	0.667

Refinement	
*R*[*F* ^2^ > 2σ(*F* ^2^)], *wR*(*F* ^2^), *S*	0.037, 0.090, 1.04
No. of reflections	5238
No. of parameters	262
No. of restraints	1
H-atom treatment	H-atom parameters constrained
Δρ_max_, Δρ_min_ (e Å^−3^)	0.21, −0.23
Absolute structure	Refined as an inversion twin
Absolute structure parameter	0.16 (9)

Computer programs: *APEX2* and *SAINT* (Bruker, 2014 ▸), *SHELXS97* and *SHELXTL* (Sheldrick, 2008 ▸), *SHELXL2014* (Sheldrick, 2015 ▸), *DIAMOND* (Brandenburg, 2010 ▸) and *publCIF* (Westrip, 2010 ▸).

## supplementary crystallographic information

### Crystal data

**Table:**

C_7_H_13_N_3_O_3_S	*D*_x_ = 1.345 Mg m^−^^3^
*M**_r_* = 219.26	Mo *K*α radiation, λ = 0.71073 Å
Orthorhombic, *P**c**a*2_1_	Cell parameters from 6335 reflections
*a* = 8.3367 (4) Å	θ = 2.5–27.8°
*b* = 10.7752 (5) Å	µ = 0.29 mm^−^^1^
*c* = 24.1016 (12) Å	*T* = 173 K
*V* = 2165.04 (18) Å^3^	Plate, colourless
*Z* = 8	0.50 × 0.14 × 0.11 mm
*F*(000) = 928	

### Data collection

**Table:**

Bruker APEXII CCD diffractometer	4655 reflections with *I* > 2σ(*I*)
φ and ω scans	*R*_int_ = 0.035
Absorption correction: multi-scan (SADABS; Bruker, 2014)	θ_max_ = 28.3°, θ_min_ = 1.7°
*T*_min_ = 0.665, *T*_max_ = 0.746	*h* = −11→11
19300 measured reflections	*k* = −14→14
5238 independent reflections	*l* = −32→31

### Refinement

**Table:**

Refinement on *F*^2^	Hydrogen site location: inferred from neighbouring sites
Least-squares matrix: full	H-atom parameters constrained
*R*[*F*^2^ > 2σ(*F*^2^)] = 0.037	*w* = 1/[σ^2^(*F*_o_^2^) + (0.0432*P*)^2^ + 0.3998*P*] where *P* = (*F*_o_^2^ + 2*F*_c_^2^)/3
*wR*(*F*^2^) = 0.090	(Δ/σ)_max_ < 0.001
*S* = 1.04	Δρ_max_ = 0.21 e Å^−^^3^
5238 reflections	Δρ_min_ = −0.23 e Å^−^^3^
262 parameters	Absolute structure: Refined as an inversion twin
1 restraint	Absolute structure parameter: 0.16 (9)

### Special details

**Table:**

Geometry. All esds (except the esd in the dihedral angle between two l.s. planes) are estimated using the full covariance matrix. The cell esds are taken into account individually in the estimation of esds in distances, angles and torsion angles; correlations between esds in cell parameters are only used when they are defined by crystal symmetry. An approximate (isotropic) treatment of cell esds is used for estimating esds involving l.s. planes.
Refinement. Refined as a 2-component inversion twin.

### Fractional atomic coordinates and isotropic or equivalent isotropic displacement parameters (Å^2^)

**Table:**

	*x*	*y*	*z*	*U*_iso_*/*U*_eq_	
S1	0.55678 (10)	0.32807 (7)	0.87717 (3)	0.0373 (2)	
S2	0.97239 (10)	0.16264 (7)	0.54545 (3)	0.0376 (2)	
O1	0.8636 (3)	0.13823 (18)	0.74085 (9)	0.0308 (5)	
O2	0.6589 (2)	0.17413 (17)	0.79576 (8)	0.0270 (4)	
O3	0.2966 (3)	0.0966 (2)	0.91570 (10)	0.0431 (6)	
O4	1.2851 (3)	0.36802 (18)	0.67471 (9)	0.0307 (5)	
O5	1.0771 (3)	0.32628 (17)	0.62210 (9)	0.0282 (5)	
O6	0.7167 (3)	0.3997 (2)	0.50123 (10)	0.0403 (6)	
N1	0.6479 (3)	0.0112 (2)	0.73350 (10)	0.0290 (5)	
H1N	0.5497	−0.0037	0.7451	0.035*	
N2	0.5009 (3)	0.1328 (2)	0.81132 (10)	0.0272 (5)	
N3	0.1571 (3)	0.2143 (2)	0.85463 (10)	0.0303 (6)	
N4	1.0729 (3)	0.4993 (2)	0.67852 (10)	0.0287 (5)	
H4N	0.9764	0.5151	0.6655	0.034*	
N5	0.9217 (3)	0.3683 (2)	0.60490 (10)	0.0261 (5)	
N6	0.5764 (3)	0.2808 (2)	0.56227 (10)	0.0291 (6)	
C1	0.7188 (4)	−0.0672 (3)	0.69115 (14)	0.0348 (7)	
H1A	0.7844	−0.0164	0.6662	0.052*	
H1B	0.6335	−0.1079	0.6699	0.052*	
H1C	0.7863	−0.1304	0.7088	0.052*	
C2	0.7298 (4)	0.1050 (2)	0.75477 (12)	0.0244 (6)	
C3	0.4493 (4)	0.2044 (3)	0.84969 (11)	0.0242 (6)	
C4	0.4197 (5)	0.3917 (4)	0.92805 (16)	0.0481 (10)	
H4A	0.3255	0.4260	0.9091	0.072*	
H4B	0.4736	0.4576	0.9490	0.072*	
H4C	0.3858	0.3259	0.9536	0.072*	
C5	0.2909 (3)	0.1659 (3)	0.87589 (13)	0.0271 (6)	
C6	0.0028 (4)	0.1835 (4)	0.88024 (16)	0.0398 (8)	
H6A	0.0171	0.1138	0.9059	0.060*	
H6B	−0.0742	0.1604	0.8513	0.060*	
H6C	−0.0376	0.2557	0.9007	0.060*	
C7	0.1526 (4)	0.3046 (3)	0.80973 (14)	0.0372 (7)	
H7A	0.1188	0.3853	0.8243	0.056*	
H7B	0.0764	0.2770	0.7813	0.056*	
H7C	0.2597	0.3122	0.7933	0.056*	
C8	1.1426 (4)	0.5816 (3)	0.71943 (13)	0.0348 (7)	
H8A	1.1834	0.5326	0.7507	0.052*	
H8B	1.0607	0.6396	0.7327	0.052*	
H8C	1.2311	0.6281	0.7026	0.052*	
C9	1.1516 (4)	0.4014 (2)	0.66055 (11)	0.0242 (6)	
C10	0.8681 (4)	0.2936 (2)	0.56845 (11)	0.0240 (6)	
C11	0.8379 (5)	0.0982 (3)	0.49430 (15)	0.0462 (9)	
H11A	0.7431	0.0641	0.5129	0.069*	
H11B	0.8928	0.0320	0.4739	0.069*	
H11C	0.8049	0.1635	0.4684	0.069*	
C12	0.7109 (4)	0.3295 (2)	0.54159 (13)	0.0267 (6)	
C13	0.5720 (4)	0.1938 (3)	0.60790 (14)	0.0366 (7)	
H13A	0.5364	0.1126	0.5944	0.055*	
H13B	0.4972	0.2238	0.6363	0.055*	
H13C	0.6795	0.1861	0.6240	0.055*	
C14	0.4231 (4)	0.3106 (4)	0.53622 (15)	0.0420 (8)	
H14A	0.4301	0.3922	0.5184	0.063*	
H14B	0.3386	0.3120	0.5645	0.063*	
H14C	0.3976	0.2476	0.5083	0.063*	

### Atomic displacement parameters (Å^2^)

**Table:**

	*U*^11^	*U*^22^	*U*^33^	*U*^12^	*U*^13^	*U*^23^
S1	0.0356 (4)	0.0368 (4)	0.0393 (4)	−0.0072 (3)	0.0091 (4)	−0.0137 (4)
S2	0.0352 (4)	0.0330 (4)	0.0447 (5)	0.0083 (3)	−0.0103 (4)	−0.0155 (4)
O1	0.0210 (10)	0.0284 (10)	0.0428 (12)	−0.0018 (9)	0.0060 (9)	−0.0027 (9)
O2	0.0212 (10)	0.0271 (10)	0.0325 (11)	−0.0017 (8)	0.0047 (9)	−0.0055 (8)
O3	0.0392 (14)	0.0478 (13)	0.0423 (13)	0.0036 (11)	0.0085 (11)	0.0196 (11)
O4	0.0246 (11)	0.0275 (10)	0.0401 (12)	−0.0007 (9)	−0.0058 (10)	0.0029 (9)
O5	0.0211 (10)	0.0257 (10)	0.0379 (12)	0.0023 (8)	−0.0061 (9)	−0.0072 (9)
O6	0.0342 (13)	0.0476 (12)	0.0393 (12)	0.0008 (11)	−0.0035 (10)	0.0185 (11)
N1	0.0227 (12)	0.0282 (11)	0.0360 (13)	−0.0003 (10)	0.0061 (10)	−0.0077 (10)
N2	0.0233 (13)	0.0265 (12)	0.0318 (13)	−0.0011 (11)	0.0054 (11)	0.0001 (10)
N3	0.0269 (13)	0.0338 (13)	0.0303 (13)	−0.0022 (11)	0.0031 (11)	0.0026 (10)
N4	0.0259 (12)	0.0261 (11)	0.0343 (12)	0.0015 (10)	−0.0078 (10)	−0.0065 (10)
N5	0.0204 (12)	0.0268 (11)	0.0310 (13)	0.0025 (10)	−0.0039 (10)	−0.0028 (10)
N6	0.0242 (13)	0.0338 (12)	0.0293 (13)	−0.0019 (10)	−0.0014 (10)	−0.0019 (10)
C1	0.0351 (17)	0.0295 (15)	0.0398 (17)	0.0018 (13)	0.0061 (14)	−0.0103 (12)
C2	0.0232 (15)	0.0219 (12)	0.0281 (14)	0.0057 (11)	0.0031 (12)	0.0023 (11)
C3	0.0265 (15)	0.0235 (13)	0.0225 (13)	0.0020 (12)	0.0005 (11)	0.0021 (11)
C4	0.047 (2)	0.052 (2)	0.045 (2)	0.0018 (18)	0.0094 (18)	−0.0225 (17)
C5	0.0276 (15)	0.0256 (13)	0.0282 (14)	−0.0014 (11)	0.0051 (13)	−0.0014 (12)
C6	0.0255 (15)	0.055 (2)	0.0388 (17)	−0.0041 (15)	0.0053 (16)	−0.0005 (16)
C7	0.0364 (18)	0.0385 (17)	0.0368 (17)	0.0014 (14)	−0.0027 (15)	0.0072 (14)
C8	0.0439 (19)	0.0293 (14)	0.0311 (15)	−0.0019 (14)	−0.0055 (14)	−0.0050 (12)
C9	0.0236 (14)	0.0225 (13)	0.0266 (13)	−0.0036 (11)	−0.0002 (12)	0.0020 (11)
C10	0.0262 (15)	0.0210 (12)	0.0249 (13)	0.0001 (12)	0.0008 (11)	0.0001 (11)
C11	0.048 (2)	0.0461 (19)	0.045 (2)	0.0003 (17)	−0.0105 (17)	−0.0213 (16)
C12	0.0277 (15)	0.0253 (12)	0.0271 (14)	0.0005 (11)	−0.0025 (13)	−0.0024 (12)
C13	0.0346 (18)	0.0368 (16)	0.0383 (18)	−0.0050 (14)	0.0050 (14)	0.0040 (14)
C14	0.0240 (15)	0.057 (2)	0.045 (2)	0.0030 (15)	−0.0041 (15)	0.0001 (16)

### Geometric parameters (Å, º)

**Table:**

S1—C3	1.737 (3)	C1—H1A	0.9800
S1—C4	1.811 (3)	C1—H1B	0.9800
S2—C10	1.748 (3)	C1—H1C	0.9800
S2—C11	1.805 (3)	C3—C5	1.521 (4)
O1—C2	1.218 (4)	C4—H4A	0.9800
O2—C2	1.371 (3)	C4—H4B	0.9800
O2—N2	1.440 (3)	C4—H4C	0.9800
O3—C5	1.216 (4)	C6—H6A	0.9800
O4—C9	1.219 (4)	C6—H6B	0.9800
O5—C9	1.378 (3)	C6—H6C	0.9800
O5—N5	1.434 (3)	C7—H7A	0.9800
O6—C12	1.233 (4)	C7—H7B	0.9800
N1—C2	1.323 (4)	C7—H7C	0.9800
N1—C1	1.451 (4)	C8—H8A	0.9800
N1—H1N	0.8800	C8—H8B	0.9800
N2—C3	1.279 (4)	C8—H8C	0.9800
N3—C5	1.334 (4)	C10—C12	1.512 (4)
N3—C7	1.455 (4)	C11—H11A	0.9800
N3—C6	1.464 (4)	C11—H11B	0.9800
N4—C9	1.315 (4)	C11—H11C	0.9800
N4—C8	1.448 (4)	C13—H13A	0.9800
N4—H4N	0.8800	C13—H13B	0.9800
N5—C10	1.272 (4)	C13—H13C	0.9800
N6—C12	1.334 (4)	C14—H14A	0.9800
N6—C13	1.445 (4)	C14—H14B	0.9800
N6—C14	1.460 (4)	C14—H14C	0.9800
			
C3—S1—C4	102.89 (16)	N3—C6—H6C	109.5
C10—S2—C11	102.61 (16)	H6A—C6—H6C	109.5
C2—O2—N2	114.5 (2)	H6B—C6—H6C	109.5
C9—O5—N5	114.6 (2)	N3—C7—H7A	109.5
C2—N1—C1	120.5 (2)	N3—C7—H7B	109.5
C2—N1—H1N	119.8	H7A—C7—H7B	109.5
C1—N1—H1N	119.8	N3—C7—H7C	109.5
C3—N2—O2	108.1 (2)	H7A—C7—H7C	109.5
C5—N3—C7	124.6 (3)	H7B—C7—H7C	109.5
C5—N3—C6	118.9 (3)	N4—C8—H8A	109.5
C7—N3—C6	116.3 (3)	N4—C8—H8B	109.5
C9—N4—C8	121.0 (3)	H8A—C8—H8B	109.5
C9—N4—H4N	119.5	N4—C8—H8C	109.5
C8—N4—H4N	119.5	H8A—C8—H8C	109.5
C10—N5—O5	108.5 (2)	H8B—C8—H8C	109.5
C12—N6—C13	124.1 (3)	O4—C9—N4	126.9 (3)
C12—N6—C14	119.2 (3)	O4—C9—O5	115.2 (2)
C13—N6—C14	116.6 (3)	N4—C9—O5	117.9 (3)
N1—C1—H1A	109.5	N5—C10—C12	116.0 (2)
N1—C1—H1B	109.5	N5—C10—S2	123.7 (2)
H1A—C1—H1B	109.5	C12—C10—S2	120.1 (2)
N1—C1—H1C	109.5	S2—C11—H11A	109.5
H1A—C1—H1C	109.5	S2—C11—H11B	109.5
H1B—C1—H1C	109.5	H11A—C11—H11B	109.5
O1—C2—N1	126.2 (3)	S2—C11—H11C	109.5
O1—C2—O2	115.7 (2)	H11A—C11—H11C	109.5
N1—C2—O2	118.1 (3)	H11B—C11—H11C	109.5
N2—C3—C5	115.3 (2)	O6—C12—N6	124.7 (3)
N2—C3—S1	124.4 (2)	O6—C12—C10	117.4 (3)
C5—C3—S1	119.9 (2)	N6—C12—C10	117.9 (3)
S1—C4—H4A	109.5	N6—C13—H13A	109.5
S1—C4—H4B	109.5	N6—C13—H13B	109.5
H4A—C4—H4B	109.5	H13A—C13—H13B	109.5
S1—C4—H4C	109.5	N6—C13—H13C	109.5
H4A—C4—H4C	109.5	H13A—C13—H13C	109.5
H4B—C4—H4C	109.5	H13B—C13—H13C	109.5
O3—C5—N3	125.1 (3)	N6—C14—H14A	109.5
O3—C5—C3	117.4 (3)	N6—C14—H14B	109.5
N3—C5—C3	117.4 (3)	H14A—C14—H14B	109.5
N3—C6—H6A	109.5	N6—C14—H14C	109.5
N3—C6—H6B	109.5	H14A—C14—H14C	109.5
H6A—C6—H6B	109.5	H14B—C14—H14C	109.5
			
C2—O2—N2—C3	179.8 (2)	S1—C3—C5—N3	93.6 (3)
C9—O5—N5—C10	178.1 (2)	C8—N4—C9—O4	0.0 (5)
C1—N1—C2—O1	3.0 (5)	C8—N4—C9—O5	179.2 (2)
C1—N1—C2—O2	−178.2 (3)	N5—O5—C9—O4	−178.6 (2)
N2—O2—C2—O1	179.9 (2)	N5—O5—C9—N4	2.1 (3)
N2—O2—C2—N1	1.0 (3)	O5—N5—C10—C12	−174.7 (2)
O2—N2—C3—C5	−173.4 (2)	O5—N5—C10—S2	0.4 (3)
O2—N2—C3—S1	−0.5 (3)	C11—S2—C10—N5	−179.2 (3)
C4—S1—C3—N2	179.3 (3)	C11—S2—C10—C12	−4.2 (3)
C4—S1—C3—C5	−8.0 (3)	C13—N6—C12—O6	176.8 (3)
C7—N3—C5—O3	175.0 (3)	C14—N6—C12—O6	0.1 (4)
C6—N3—C5—O3	−0.1 (5)	C13—N6—C12—C10	−1.4 (4)
C7—N3—C5—C3	−2.4 (4)	C14—N6—C12—C10	−178.1 (3)
C6—N3—C5—C3	−177.5 (3)	N5—C10—C12—O6	85.8 (3)
N2—C3—C5—O3	89.3 (3)	S2—C10—C12—O6	−89.5 (3)
S1—C3—C5—O3	−84.0 (3)	N5—C10—C12—N6	−95.9 (3)
N2—C3—C5—N3	−93.1 (3)	S2—C10—C12—N6	88.8 (3)

### Hydrogen-bond geometry (Å, º)

**Table:**

*D*—H···*A*	*D*—H	H···*A*	*D*···*A*	*D*—H···*A*
N1—H1*N*···O1^i^	0.88	2.13	2.871 (3)	142
N4—H4*N*···O4^ii^	0.88	2.04	2.794 (3)	142
C4—H4*B*···O6^iii^	0.98	2.54	3.075 (4)	114
C6—H6*B*···O2^iv^	0.98	2.60	3.518 (4)	156
C7—H7*B*···O1^iv^	0.98	2.52	3.431 (4)	155
C11—H11*B*···O3^v^	0.98	2.53	3.042 (4)	113
C13—H13*B*···O4^iv^	0.98	2.53	3.440 (4)	155
C14—H14*B*···O5^iv^	0.98	2.59	3.554 (4)	168

Symmetry codes: (i) *x*−1/2, −*y*, *z*; (ii) *x*−1/2, −*y*+1, *z*; (iii) −*x*+1, −*y*+1, *z*+1/2; (iv) *x*−1, *y*, *z*; (v) −*x*+1, −*y*, *z*−1/2.
